# Ultra-structural defects cause low bone matrix stiffness despite high mineralization in osteogenesis imperfecta mice^☆^

**DOI:** 10.1016/j.bone.2012.03.007

**Published:** 2012-06

**Authors:** Maximilien Vanleene, Alexandra Porter, Pascale-Valerie Guillot, Alan Boyde, Michelle Oyen, Sandra Shefelbine

**Affiliations:** a Department of Bioengineering, Imperial College London, London,SW7-2AZ, UK; b Department of Materials, Imperial College London, London, SW7-2AZ, UK; c Institute of Reproductive and Developmental Biology, Imperial College London, London, W12 0NN, UK; d Dental Physical Sciences, Barts and The London School of Medicine and Dentistry, QMUL, London, E1 4NS, UK; e Department of Engineering, Cambridge University, Cambridge, CB2-1PZ, UK

**Keywords:** Osteogenesis imperfecta, Bone matrix, Stiffness, Mineralization, Mouse model

## Abstract

Bone is a complex material with a hierarchical multi-scale organization from the molecule to the organ scale. The genetic bone disease, osteogenesis imperfecta, is primarily caused by mutations in the collagen type I genes, resulting in bone fragility. Because the basis of the disease is molecular with ramifications at the whole bone level, it provides a platform for investigating the relationship between structure, composition, and mechanics throughout the hierarchy. Prior studies have individually shown that OI leads to: 1. increased bone mineralization, 2. decreased elastic modulus, and 3. smaller apatite crystal size. However, these have not been studied together and the mechanism for how mineral structure influences tissue mechanics has not been identified. This lack of understanding inhibits the development of more accurate models and therapies. To address this research gap, we used a mouse model of the disease (*oim*) to measure these outcomes together in order to propose an underlying mechanism for the changes in properties. Our main finding was that despite increased mineralization, *oim* bones have lower stiffness that may result from the poorly organized mineral matrix with significantly smaller, highly packed and disoriented apatite crystals. Using a composite framework, we interpret the lower *oim* bone matrix elasticity observed as the result of a change in the aspect ratio of apatite crystals and a disruption of the crystal connectivity.

## Highlights

► We have investigated osteogenesis imperfecta mice (*oim*) bone matrix. *► oim* mice have lower matrix stiffness and higher mineralization than wild type mice. ► Poor correlation found between stiffness and mineralization. ► Changes in apatite crystal structure and connectivity may play a role the degradation of material properties despite higher density.

## Introduction

Bone is a heterogeneous and complex material with structural and mechanical properties organized from the organ scale to the molecule scale in a hierarchical framework [1]. A positive correlation between bone mineral density and elastic modulus has been established at the macroscopic (whole bone) scale [2] and is commonly used in assessing fracture risk, diagnosing osteoporosis, and measuring the efficacy of therapies [3–5]. However, at the microscopic (matrix) scale, this relationship is less clear as correlations of bone matrix mechanical properties with the mineral content are weaker than macroscopic correlations [6–8]. Previous studies have highlighted the importance of the collagen matrix organization and content on microscopic mechanical properties in calcified cartilage, subchondral bone, and cortical bone [7,9].

Osteogenesis imperfecta (OI or brittle bone disease) is primarily caused by mutations in collagen type 1 genes and results in bone fragility [10–13]. OI provides an interesting platform for investigating how alterations at the molecular level cause changes in structure and mechanics throughout the hierarchy of bone. In the present investigation, we used the *oim* model, in which the mice do not express col1-α2 protein and have homotrimeric collagen1-(α1)_3_ instead of the normal heterotrimer helix. These mice have extreme bone fragility, mimicking moderate to severe OI in humans. At the macroscopic scale (whole bone), published measures of *oim* bone intrinsic elastic properties are contradictory, either greater than [14,15] or equivalent to [16,17] or lower than [18,19] normal wild type mice bone. Such discrepancies can be explained by the difference in the testing techniques used and their limitations to access accurately the intrinsic bone elasticity.

At the microscopic (matrix) scale, *oim* bone is mostly composed of woven tissue [20] with unorganized collagen fibers, a high mineral/protein content ratio [21,22] and a high porosity [23]. This results in a low bone mineral density (content) measured by DXA on the whole bone level [24]. At the collagen/apatite scale (ultrastructure with nm length scale), *oim* bone apatite crystals are small and not well aligned [25,26] and their crystallinity and chemical composition is altered [21,22].

Numerous studies have examined the macroscopic mechanical properties of *oim* bone [15,16,18,19], the microscopic matrix mineral content [14,21,22,24], or the ultra-structure [25]. Only Grabner et al. investigated both mechanics and mineralization at the microscopic scale [26]. The mechanical measures were however limited to measures of the Vicker's micro-hardness, which provides no information on the bone matrix elastic properties. No previous study has examined the multi-scale changes in mineral structure, density, and elastic modulus in *oim* bone in order to explain how changes at the molecular level are translated into altered mechanical behavior at larger length scales.

The objective of this study was to determine the multi-scale material properties in *oim* bone, and in particular correlations between local tissue mineralization and elastic modulus at the microscopic (μm) scale. We used 3-point bending to estimate whole bone elastic modulus, quantitative backscattered electron microscopy (qBSEM) to quantify the amount of bone matrix mineral, nanoindentation to measure the bone matrix elastic and plastic properties, and transmission electron microscopy (TEM) to examine the apatite crystals size and organization. We propose a mechanistic interpretation linking the mechanical and structural properties observed at the matrix scale into a common composite material framework. With an understanding of how structural changes influence mechanical behavior, appropriate pharmaceutical therapies might be targeted to address particular critical deficiencies in bone.

## Material and method

### Specimens

Wild type B6C3Fe-a/a-+/+ mice (WT, 8♀, 7♂) and pathologic B6C3Fe-a/a-Col1a2*Oim*/*Oim* mice (*oim*, 8♀, 12♂) were culled at 8 weeks-old and long bones were collected, cleaned of soft tissues and stored in gauze soaked with a phosphate buffered saline solution at − 18 °C.

### Macroscopic mechanical testing

For each specimen, the right femurs were tested until fracture by 3-point bending using a standard materials testing machine (5866 Instron). The femurs were loaded at the mid-diaphysis in the anterior–posterior direction with a deflection rate of 50 m/s. Force–deflection curves were analyzed with a custom program (Matlab, MathWorks) to measure the bending stiffness (S, N.mm) and ultimate force (F_ult_, N). The intrinsic elastic modulus E (MPa) and ultimate stress σ_ult_ (MPa), were calculated using the standard beam theory [27] and geometrical data previously measured from μCT images of the femurs [17].

### Specimen preparation for microscopic analyses

In order to analyze the bone matrix mineralization, mechanical properties and intra-specimen variations at the microscopic scale, tibiae were collected from four mice (2 males, 2 females), randomly selected from the wild type group and from the *oim* group. The bones were fixed in 70% ethanol (1 week), dehydrated using a graded ethanol series (70, 80, 95 and 99% for 48 h in each), and substituted with xylene (24 h). The specimens were then infiltrated for 48 h in two successive changes of pure methyl methacrylate (MMA) replaced by two changes of MMA + α-azo-iso-butyronitrile (24 h) and finally polymerized slowly at 37 °C (all chemicals purchased from VWR, UK). The tibiae were sectioned transversally at the mid-diaphysis with a low speed diamond saw (Isomet, Buehler GmbH, Germany) and the cross-sections were ground with increasingly finer grades of carbide papers (from P500 to P4000) and finally polished with diamond slurry (diameter: 0.25 and 0.05 μm).

### Quantitative backscattered electron microscopy (qBSEM)

The tibia mid-diaphyseal cross-sections were carbon coated and analyzed using qBSEM in an EVO®MA15 scanning electron microscope (Zeiss UK Ltd., UK) operated at 20 kV, at a working distance of 13 mm, and a beam current of 0.5 nA. The qBSEM digital images were recorded with a nominal magnification of 137 × (field width: 2.133 mm, pixel size: 1.04 μm). The image backscattered electron (BSE) current signal (digitized in gray levels) were standardized against the BSE signals of monobromo and monoiodo dimethacrylate standards which span the signal range found for mineralized tissues: 0 (black, monobrom) representing osteoid and 255 (white, monoiod) representing highly mineralized bone [28,29]. To facilitate visualization, the gray-level range was also divided into 8 equal size classes (1–32, 33–64, 65–96, 97–128, 129–160, 161–192, 193–224, 225–255), representing no mineralization (class 1) to very high bone mineralization (class 8). The distribution of pixels into the different bone mineralization classes was then calculated and provides an estimate of the amount and distribution of bone mineral within a sample. For numerical analysis, each cross section image was automatically divided by a custom Matlab program into 12 areas corresponding to the periosteal, mid-cortex and endosteal sectors of the anterior, lateral, posterior and medial cross section quadrants. The mean pixel gray-level value in each sector was then calculated as an estimate of the mean amount of bone mineral in this sector.

### Nanoindentation

Nanoindentation tests were conducted on the same tibia mid-diaphyseal cross-sections to a maximum load of 8 mN at a constant loading rate of 800 μN/s in the longitudinal axis using the TI700 UBI (Hysitron, MN, USA) with a Berkovich diamond tip. A total of 60 indents were performed per specimen around the tibial cross section: five indents in the periosteal, mid-cortex and endosteal sectors of the anterior, lateral, posterior and medial quadrants of the cross-section. Time–depth–force data during unload were fitted with a viscous–elastic–plastic (VEP) mathematical model [30,31] in order to determine the plane-strain elastic modulus (E'), the resistance to plastic deformation (H) and the indentation viscosity (η), using Origin 8 software (Originlab Corp., MN, USA). The bone matrix compressive elastic modulus (E_nano_) was calculated as E' = E_nano_/(1 − ν^2^) with Poisson's ratio ν = 0.3 [32]. The resistance to plastic deformation H is an estimation of the purely plastic deformation occurring during loading and is independent from the tissue elasticity, contrary to the contact hardness (H_c_) usually measured using nanoindentation [33]. Viscous deformation was found negligible compared to elastic and plastic deformations (< 2% of total deformation) and was not considered further.

### Transmission electron microscopy (TEM) imaging

To investigate the apatite crystal nano-structural organization, humeri were collected from the four mice (2 males, 2 females) randomly selected from each groups. The humeri were prepared using an anhydrous embedding protocol in order to optimally preserve mineral chemistry and structure. This protocol was previously used on dentine and enamel for TEM examination [34]. The bones were first dehydrated separately in ethylene glycol (24 h), then washed in 100% ethanol 3 times for 10 min in each, followed by three changes of acetonitrile, a transitional solvent for 15 min in each. Specimens were then infiltrated separately with epoxy resin for a total of 11 days. The epoxy resin was prepared by mixing 12 g Quetol651, 15.5 g nonenylsuccinic anhydride (NSA), 6.5 g methylnadic anhydride (MNA), and 0.6 g benzyldimethylamine (BDMA) (Agar Scientific, Essex, UK). The samples were placed successively in a 1:1 then 3:1 volume ratio of resin:acetonitrile solutions for 24 h in each. Samples were then infiltrated with 100% resin under vacuum, changed every 24 h, for eight successive days. On the 12th day, samples were placed separately in truncated capsules with fresh resin and cured at 60 °C for 48 h.

Resin embedded specimens were then sectioned longitudinally using a Powertome XL ultramicrotome (RMC products by Boeckeler® instruments Inc., AZ, USA) in slices of 50 to 70 nm thickness with a ultra 45° Diatome diamond blade (Diatome AG, Switzerland) and collected immediately on Holey carbon coated copper grids (square mesh 300) for TEM observation. Sample slices were imaged using a JEOL 2010 TEM microscope operated at 120 kV at 25 to 60K × magnification to observe the apatite crystals. To estimate the crystal size, we have used the method described by Porter et al. [34]. The apatite crystal thickness (short axis of the apatite crystal plate side) was measured for crystals that could be clearly distinguished in four TEM micrographs per specimens at 60K × magnification using ImageJ software.

### Statistical analyses

All analyses were performed with using SPSS 17.0 software (SPSS Inc., IL, USA). The level of statistical significance of the tests was set at 5%. Macroscopic mechanical properties of bone were compared using multi-variable analysis of variance (ANOVA).

As substantial regional variations of the tissue properties within a bone have been previously reported [7,35], we sampled each specimen thoroughly (60 indents) to assess and correlate the local bone tissue properties (rather than perform a few indents on a large number of specimens). Multifactor analyses of variance (ANOVA) tests were run for nanoindentation and qBSEM data with mice gender and type, cross section quadrants and cortex sectors as factors and specimen as covariate to account for the low number of specimens tested. For TEM measures, ANOVA tests were run with mice gender and type as factors and specimen as covariate. ANOVA were followed by post hoc Bonferroni tests. Correlations between bone matrix mechanical properties and bone mineral content were analyzed using Pearson's correlation (level of significance: 5%).

## Results

### Macroscopic mechanical properties

The bending stiffness S and ultimate force F_ult_ were significantly lower in the *oim* mice compared to the wild type mice (p < 0.001). The calculated elastic modulus (E) was not significantly different between *oim* and wild type animals (p > 0.05) while the ultimate stress (σ_ult_) was lower in *oim* mice compared to wild type mice (p < 0.001) (Table 1).

### Quantitative backscattered electron microscopy

**Fig. 1:**
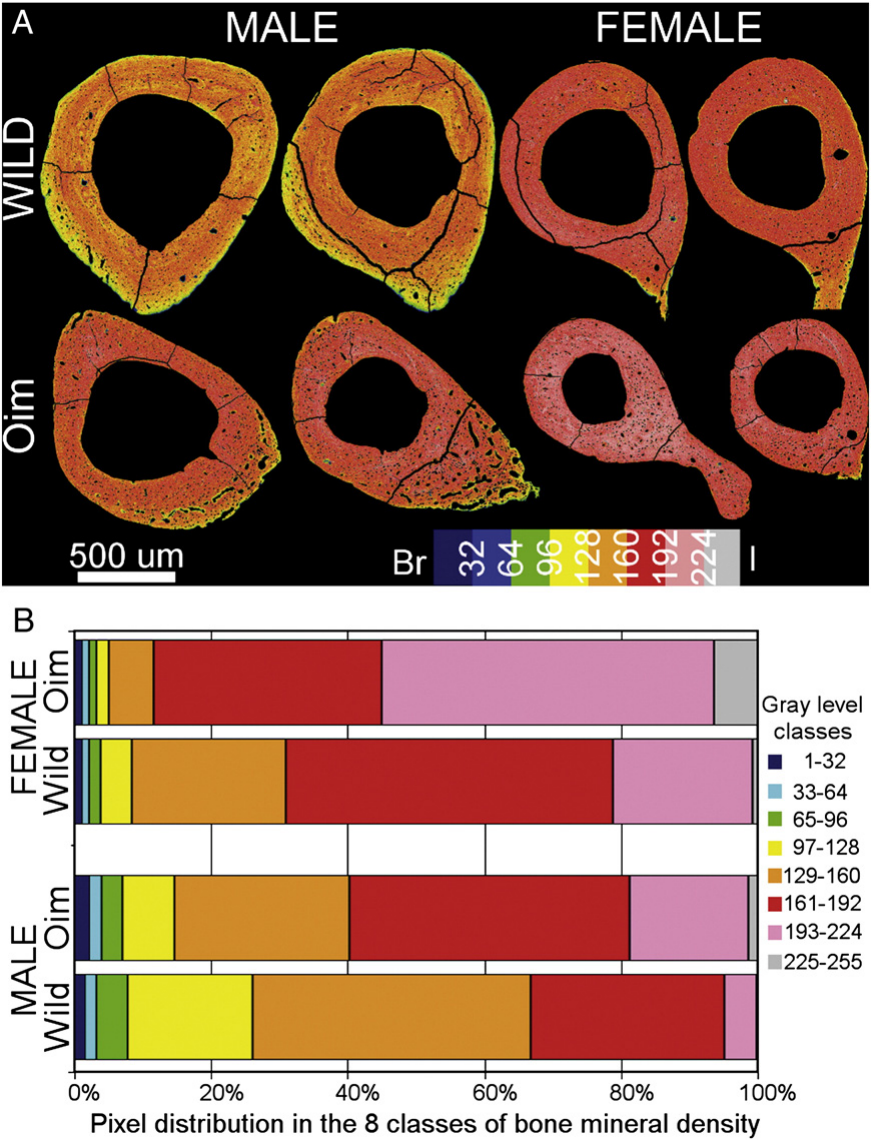
Bone matrix mineralization of the *oim* and wild type mice tibial cross-sections analyzed by quantitative backscattered electron microscopy: (A) qBSE micrographs of the tibial cross-sections displaying the 8 classes of bone mineralization (gray scale) represented with pseudo colors (1–32: dark blue, 33–64:light blue, 65–96: green, 97–128: yellow, 129–160: orange, 161–192: red, 193–224: pink, 225–255:gray). (B) Pixel distributions into the 8 classes of bone mineralization for the *oim* and wild type male and female specimens. The greater red/pink area in *oim* bone indicates higher mineral density.

The qBSEM images taken from each *oim* and wild type mice tibiae and the distribution of the pixels into the 8 different classes (gray-level) of bone mineralization are illustrated in Fig. 1A and B. *Oim* mice had a significantly higher amount of mineral than the wild type mice (p < 0.001). The amount of bone mineral was higher in females than in males (p < 0.001).

### Nanoindentation testing

The mean elastic modulus E_nano_ was significantly lower in *oim* (33.8 ± 5.5 GPa) than in wild type mice (41.8 ± 2.9 GPa) (p < 0.001). The bone matrix resistance to plastic deformation H was slightly but significantly larger in the *oim* mice compared to wild type mice (2.07 ± 0.09 GPa and 1.99 ± 0.12 GPa respectively, p < 0.05).

### Transmission electron microscopy

**Fig. 2:**
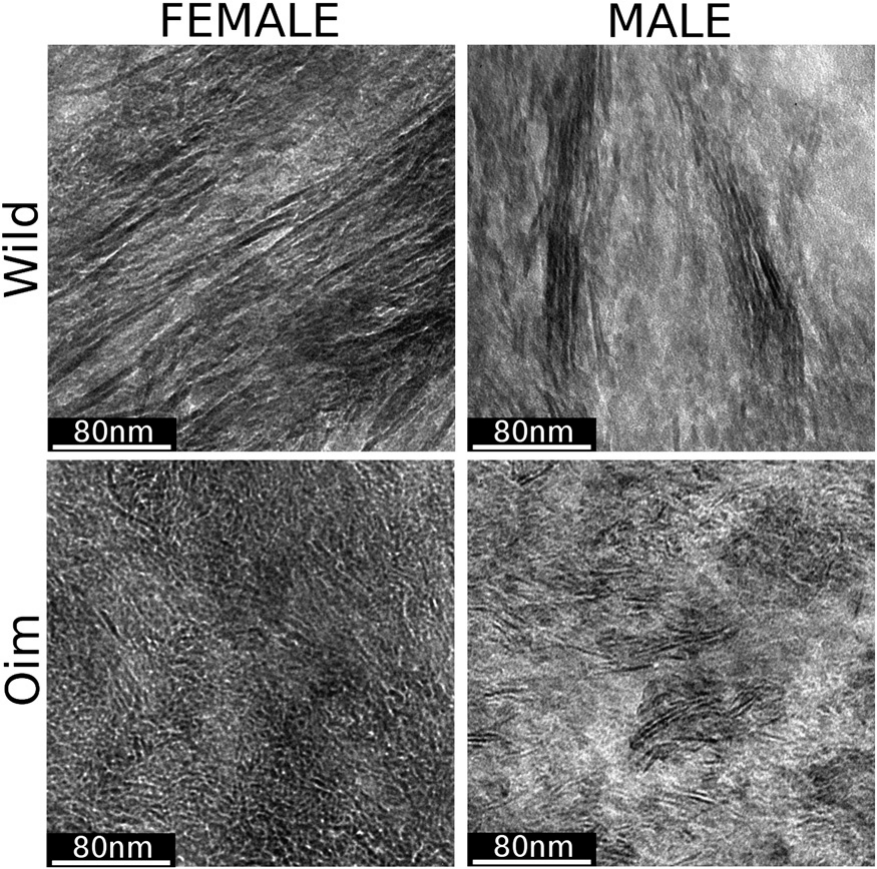
Transmission electron micrographs obtained from cortical bone matrix of wild type and *oim* mice humerus (magnification 60K ×). The black structures are the apatite crystals observed on edge. Wild type specimen displays large well-aligned crystals while *oim* specimens exhibit small disorganized crystals.

Apatite mineral in the wild type bone matrix appeared to be well aligned, needle-like crystals (when observed from the side) while in *oim* bone matrix, the crystals appeared smaller and disorganized (Fig. 2). The thickness of the apatite crystals was significantly smaller (p < 0.001) in the *oim* mice than in the wild type mice (Table 1).

### Correlation of bone matrix elasticity and mineralization

**Fig. 3:**
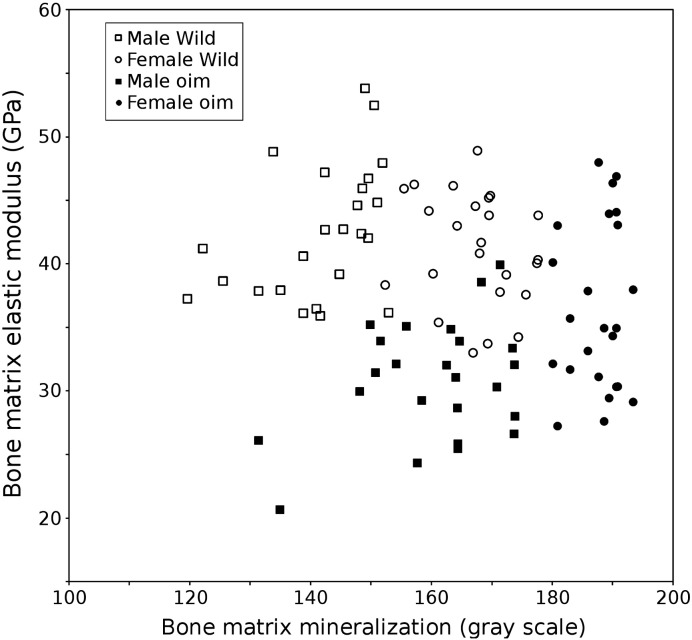
Bone matrix elasticity (vertical axis) plotted against the bone mineralization (horizontal axis) obtained from wild type and *oim* female and male mice. No strong significant correlation can be observed. However, *oim* has lower elasticity despite higher mineralization compared to wild type bone matrix. Females also display higher mineralization than males for both *oim* and wild type group with no increase of elasticity.

For both wild type and *oim* mice, the bone matrix elastic modulus averaged in each sector around the tibia cross-section was plotted against the bone matrix mineral amount measured at the same location (Fig. 3).

Bone matrix mineral amount and elastic modulus were not correlated within each specimen (Pearson's r median = 0.434, minimum = 0.083, maximum = 0.557, p > 0.05 for all specimens) for both wild type and *oim* groups (Fig. 3). In both wild type and *oim* groups, females had a higher mineralization with no increase in modulus.

When mice were investigated per type, a weak but significant correlation was observed in the *oim* group (Pearson's r = 0.482, p < 0.01) while the wild type group exhibited no correlation (Pearson's r = − 0.007, p > 0.05).

## Discussion

Much research at the macro-scale has assumed that an increase in bone mineral density is associated with increased bone stiffness. Indeed, the gold standard for measuring therapeutic benefits of pharmaceutical therapies is measuring bone mass typically with DEXA or pQCT. Here we show in the extreme example of the *oim* model that macro-scale properties do not accurately reflect the mechanics at smaller length scales and that increases in bone matrix mineralization are not always associated with increased bone elastic properties. Osteogenesis imperfecta provides an interesting model to explore the mineral/protein relationship in the bone matrix composite, as defects in the collagen influence the structure and mechanics at multiple length scales.

At the macroscopic scale, *oim* bone was weak (decrease of F_ult_ and σ_ult_) and brittle (little post-yield deformation) as expected. The calculated elastic moduli of *oim* and wild type bone were not significantly different and displayed a very high variability (16.8% and 10.8% respectively). This finding, in combination with the discrepancy observed in the previous 3 point bending tests [14–16], illustrates that the assumptions required in the beam theory (pure bending, constant bone cross-section and homogeneous, isotropic bone material properties) actually over-simplify the bone properties and may not accurately capture the intrinsic bone matrix elasticity as noted by previous studies [36]. In addition, the whole bone estimates of modulus include the effects of porosity, which is significantly increased in *oim*, thereby providing an overall modulus that includes the matrix and the voids. This justifies an investigation of bone properties at a smaller scale with more dedicated techniques for determining matrix mechanical properties.

When measuring the bone properties at the micron length scales, it is not feasible to maintain large sample sizes particularly when the variation of properties within a sample has equal (or even greater) variance than between samples. To preclude biasing our measures at higher length scale, we chose the tested samples randomly from the wild type and *oim* groups and assessed how local variations in mineralization affected local elastic properties within a bone. At the microscopic (matrix) scale, nanoindentation revealed a decrease of elasticity and a slight increase of the resistance to plastic deformation (i.e. less plastic deformation) in the *oim* bone matrix compared to wild type mice. Our local nanoindentation results are comparable to the findings of Mehta et al. who also measured a decrease in elastic modulus in *oim* using ultrasound critical-angle reflectometry [19]*.* It should be noted that it was necessary to dehydrate and fully infiltrate our samples with PMMA for qBSEM analysis. Infiltration and high loading rate, however, affect nanoindentation measures by increasing the stiffness of the sample [37,38], which explains our relatively high, but not unreasonable values for mouse bone [36,39]. Moreover, as the same specimen preparation and indentation protocols were used on both wild type and oim specimens, the impact on bone matrix properties should be equivalent on both groups and should not affect the relative difference between the two. The differences between whole bone elastic modulus values (~ 7 GPa) and matrix level elastic modulus values (~ 30–35 GPa) are in line with the findings of other studies [36] and result primarily from beam theory simplifications at the whole bone level, porosity (included at the whole bone scale but not at the microscopic scale), and the sample preparation used for the nanoindentation protocol.

Quantitative backscattered analysis revealed a higher bone matrix mineralization in the *oim* bones compared to their wild type counterpart (as illustrated by more red/pink pixels in *oim* mice in Fig. 1). In both wild type and *oim* groups, females displayed higher mineralization with no increase in elastic modulus compared to their male counterpart. Similarly, compared to wild type mice, the bone matrix of *oim* mice was more mineralized but displayed a lower average elastic modulus. This implies that the “extra” mineral is not mechanically contributing to matrix elastic properties. While such observations on *oim* matrix mineralization are in agreement with the literature [17,19,21,26], this is the first time that the bone matrix elasticity, plasticity and mineralization were examined together at the microscopic scale. These results can help to explain how matrix properties result in bone brittleness at the macroscopic scale. For a same amount of energy deployed during a load, while the wild type bone matrix remains in the elastic domain, the *oim* bone matrix will reach the plastic domain where its higher resistance to plastic deformation does not allow further plastic deformation, triggering the catastrophic fracture of the bone and explaining the increased bone brittleness.

To investigate the structural features causing the bone matrix decrease in elastic modulus despite high mineralization, we examined the crystal structure using transmission electron microscopy (TEM). To our knowledge, this is the first time that TEM has been used to assess crystal size, structure, and organization in *oim* bone. Our TEM images revealed that the apatite crystals in the *oim* bone matrix were significantly smaller, more tightly packed and not as well aligned as the wild type which is in agreement with previous small-angle X-ray scattering observations [25,26]. The extremely tight packing of the small apatite crystals may explain the high mineralization of the *oim* bone matrix. The disorganization of crystals in *oim* mice may be partially explained by the difference of bone tissue fabrics. Indeed, *oim* matrix is composed of woven bone with disorganized collagen fibers [20] while wild type bone is made of lamellar bone with well-aligned collagen fibers and apatite crystals. The fibrous network is critical in providing a template for apatite crystallization and therefore defining crystal structure and organization. Our results suggest that changes in elastic properties may be caused by the altered crystal structure. Our TEM images show that in addition to the involvement of the disorganized collagen fibers, crystals are more randomly oriented within the fibers in *oim* bone. The altered mineral ultra-structure is likely the consequence of the homotrimeric nature of the collagen helix, which is known to have detrimental effects on procollagen helix folding, collagen fibril packing, and collagen cross-linking [40–44].

**Fig. 4:**
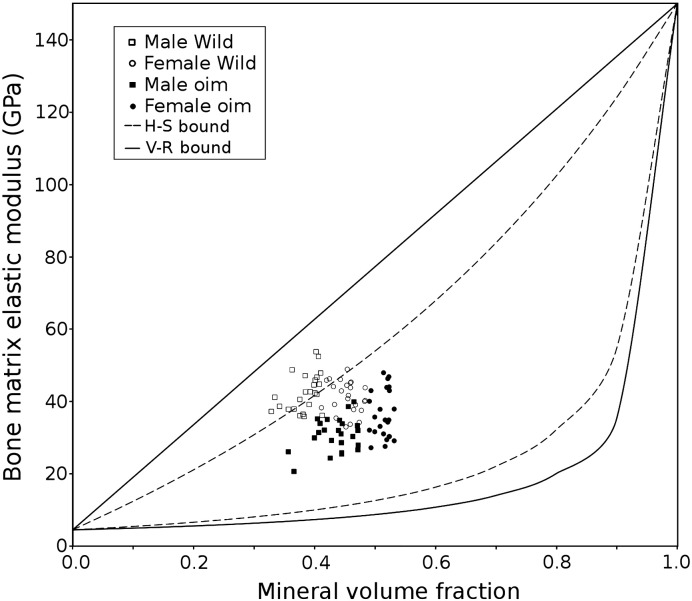
Bone matrix elasticity plotted against bone matrix mineral volume fraction obtained from the wild type and *oim* mice. The lines correspond to the bounds of the Hashin–Shtrickman composite model (dotted lines) and of the Voigt–Reuss composite model (solid lines). The limits are set by E_mineral_ = 150 GPa (the modulus of pure hydroxyapatite [50] and E_matrix_ = 4.5 GPa (the modulus of the embedding resin).

We observed a poor correlation between the elasticity and mineralization of the bone matrix in both wild type and *oim* mice. This poor correlation is in agreement with the recent micro-scale investigations performed in human cortical bones [7], articular calcified tissues from human and horse (healthy and pathologic) [9,45,46], and across species [8]. To provide a mechanistic explanation at the lowest level of the bone architecture, we interpreted our findings in the framework of the composite material mechanics, modeling bone matrix as a composite of soft (collagen) and stiff (mineral) phases. Such approaches have been considered since the 1960's [47] to compute bone elasticity from the elastic moduli and the volume fractions of its protein and mineral components. Very briefly, two main composite frameworks can be considered to provide some relationship between bone elasticity and mineral volume fraction: the aligned fiber composite (Voigt–Reuss; V–R bounds) and the spherical particle composite (Hashin–Shtrikman; H–S bounds) [8]. The V–R bounds give upper and lower modulus bounds for a composite made of stiff continuous “fibers” in a soft matrix tested respectively in directions parallel and orthogonal to the aligned fibers direction. The H–S bounds provided upper and lower boundaries for composites respectively made of a hard mineral matrix with soft protein inclusions and made of a soft matrix with hard mineral inclusions. In order to interpret our finding in this composite framework, we converted our bone qBSEM gray values into mineral volume fraction (Vf) values [8] despite the simplifying assumptions necessarily made on density and volume fraction calculation. Plotting bone matrix elasticity against the estimated mineral volume fraction (Fig. 4) shows most data are toward the upper H–S bound which would suggest that the apatite matrix is acting as a mechanically rigid matrix with soft protein inclusions. This is in accordance with other studies that have modeled the bone matrix as a mineral continuous phase reinforced with “compliant” collagenous fiber inclusions [48,49]. However, our measures display a wide range of elasticity for a narrow range of mineral volume fraction (0.37 to 0.52) and with *oim* data having lower stiffness despite having a higher mineral volume fraction. Oyen et al. [8] have also observed such a wide range of elasticity values despite equivalent mineral volume fractions and concluded that no single relation could be found to estimate the bone elasticity from its mineral composition. This large variation of bone matrix elasticity at fixed mineral composition can be explained by introducing more finely defined ultra-structural features into the composite model. In the context of a stiff continuous (or partially continuous) mineral matrix laid upon a collagen scaffold, finite element studies of the discrete ultra-structure have shown that the connectivity of the crystal particles forming the mineral phase strongly influence the bone composite modulus (at a constant mineral volume fraction) [50,51]. This crystal connectivity is related to the crystal shape (aspect ratio), orientation and arrangement, which is most likely dictated by the organization and quality of the collagen network. Here we focus on the compressive elastic properties of the matrix (nanoindentation), which are primarily related to the mineral phase. We anticipate that the altered collagen structure plays an important role in the plastic behavior of the matrix. Thus, the short, poorly arranged and tightly packed apatite crystals seen in our TEM images of *oim* bone is a consequence of the collagen alterations and may explain why *oim* modulus values are below wild type despite the increased mineralization. The composite framework allows us to examine how changes in the ultra-structure (protein/mineral structure) can alter the modulus independent of mineral fraction.

## Conclusion

We observed no correlation between the bone mineralization and stiffness at the microscopic scale either in the *oim* or in the wild type mice. This has important implications in bone pathologies and the therapeutic strategies developed to counter their effects. Therapies that promote apposition and accumulation of hyper-mineralized bone tissue, may have the limitation of accumulating bone with poor structural and mechanical properties with possible long term negative effects [52,53]. As available clinical radiographic techniques are limited in their measure of bone “quality”, it should be of great interest to develop and validate testing techniques that allow the mechanical investigation of tissue and matrix properties in the clinic.

## Figures and Tables

**Table 1:** Mechanical properties measured by three point bending (E, σ_ult_, MPa) and nanoindentation techniques (E_nano_, H, GPa) and apatite crystal thickness (nm) measured by TEM on wild type and *oim* mice cortical bone (values displayed are mean value ± standard deviation; n is the number of specimen in the group).

	Wild type	*oim*	n	p value
E (MPa)	6935.9 ± 748.9	6996.0 ± 1175.1	15	p > 0.05
σ_ult_ (MPa)	122.9 ± 8.6	90.0 ± 34.6	15	P < 0.001
E_nano_ (GPa)	41.8 ± 2.9	33.8 ± 5.4	4	p < 0.001
H (GPa)	1.99 ± 0.12	2.07 ± 0.09	4	p < 0.05
Apatite thickness (nm)	2.6 ± 0.4	1.7 ± 0.2	4	p < 0.001

## References

[1] Rho J.Y., Kuhn-Spearing L., Zioupos P. (1998). Mechanical properties and the hierarchical structure of bone. Med Eng Phys.

[2] Currey J.D. (1984). Effects of differences in mineralization on the mechanical properties of bone. Philos Trans R Soc Lond.

[3] Ammann P., Rizzoli R. (2003). Bone strength and its determinants. Osteoporos Int.

[4] Martin R.B. (1991). Determinants of the mechanical properties of bones. J Biomech.

[5] Turner C.H. (2002). Biomechanics of bone: determinants of skeletal fragility and bone quality. Osteoporos Int.

[6] Hoc T., Henry L., Verdier M., Aubry D., Sedel L., Meunier A. (2006). Effect of the microstructure on the mechanical properties of Haversian cortical bone. Bone.

[7] Zebaze R.M.D., Jones A.C., Pandy M.G., Knackstedt M.A., Seeman E. (2011). Differences in the degree of bone tissue mineralization account for little of the differences in tissue elastic properties. Bone.

[8] Oyen M.L., Ferguson V.L., Bembey A.K., Bushby A.J., Boyde A. (2008). Composite bounds on the elastic modulus of bone. J Biomech.

[9] Gupta H.S., Schratter S., Tesch W., Roschger P., Berzlanovich A., Schoeberl T. (2005). Two different correlations between nanoindentation modulus and mineral content in the bone–cartilage interface. J Struct Biol.

[10] Rauch F., Glorieux F.H. (2004). Osteogenesis imperfecta. Lancet.

[11] Traub W., Arad T., Vetter U., Weiner S. (1994). Ultrastructural studies of bones from patients with osteogenesis imperfecta. Matrix Biol.

[12] Jones S.J., Glorieux F.H., Travers R., Boyde A. (1999). The microscopic structure of bone in normal children and patients with osteogenesis imperfecta: a survey using backscattered electron imaging. Calcif Tissue Int.

[13] Forlino A., Cabral W.A., Barnes A.M., Marini J.C. (2011). New perspectives on osteogenesis imperfecta. Nat Rev Endocrinol.

[14] Miller E., Delos D., Baldini T., Wright T., Pleshko Camacho N. (2007). Abnormal mineral–matrix interactions are a significant contributor to fragility in *oim*/*oim* bone. Calcif Tissue Int.

[15] Misof B.M., Roschger P., Baldini T., Raggio C.L., Zraick V., Root L. (2005). Differential effects of alendronate treatment on bone from growing osteogenesis imperfecta and wild-type mouse. Bone.

[16] McCarthy E.A., Raggio C.L., Hossack M.D., MilIer E.A., Jain S., Boskey A.L. (2002). Alendronate treatment for infants with osteogenesis imperfecta: demonstration of efficacy in a mouse model. Pediatr Res.

[17] Vanleene M., Saldanha Z., Cloyd K.L., Jell G., Bou-Gharios G., Bassett J.H.D. (2011). Transplantation of human fetal blood stem cells in the osteogenesis imperfecta mouse leads to improvement in multiscale tissue properties. Blood.

[18] McBride D.J., Sahapiro J.R., Dunn M.G. (1998). Bone geometry and strength measurements in aging mice with the oim mutation. Calcif Tissue Int.

[19] Mehta S.S., Antich P.P., Landis W.J. (1999). Bone material elasticity in a murine model of osteogenesis imperfecta. Connect Tissue Res.

[20] Chipman S.D., Sweet H.O., McBride D.J., Davisson M.T., Marks S.C., Shuldiner A.R. (1993). Defective pro alpha 2(I) collagen synthesis in a recessive mutation in mice: a model of human osteogenesis imperfecta. Proc Natl Acad Sci U S A.

[21] Camacho N.P., Landis W.J., Boskey A.L. (1996). Mineral changes in a mouse model of osteogenesis imperfecta detected by Fourier transform infrared microscopy. Connect Tissue Res.

[22] Camacho N.P., Hou L., Toledano T.R., ILG W.A., Brayton C.F., Raggio C.L. (1999). The material basis for reduced mechanical properties in *oim* mice bones. J Bone Miner Res.

[23] Carriero A., Doube M., Levchuk A., Schneider P., Muller R., Shefelbine S.J. (2011). Cortical tissue porosity of brittle osteogenesis imperfecta bone.

[24] Phillips C.L., Bradley D.A., Schlotzhauer C.L., Bergfeld M., Libreros-Minotta C., Gawenis L.R. (2000). *Oim* mice exhibit altered femur and incisor mineral composition and decreased bone mineral density. Bone.

[25] Fratzl P., Paris O., Klaushofer K., Landis W.J. (1996). Bone mineralization in an osteogenesis imperfecta mouse model studied by small-angle X-ray scattering. J Clin Invest.

[26] Grabner B., Landis W.J., Roschger P., Rinnerthaler S., Peterlik H., Klaushofer K. (2001). Age-and genotype-dependence of bone material properties in the osteogenesis imperfecta murine model (*oim*). Bone.

[27] Schriefer J.L., Robling A.G., Warden S.J., Fournier A.J., Mason J.J., Turner C.H. (2005). A comparison of mechanical properties derived from multiple skeletal sites in mice. J Biomech.

[28] Boyde A., Travers R., Glorieux F.H., Jones S.J. (1999). The mineralization density of iliac crest bone from children with osteogenesis imperfecta. Calcif Tissue Int.

[29] Kingsmill V.J., Boyde A. (1998). Variation in the apparent density of human mandibular bone with age and dental status. J Anat.

[30] Oyen M.L., Cook R.F. (2003). Load-displacement behaviour during sharp indentation of viscous–elastic–plastic materials. J Mater Res.

[31] Oyen M.L., Ko C.-C. (2007). Examination of local variations in viscous, elastic, and plastic indentation responses in healing bone. J Mater Sci Mater Med.

[32] Rho J.Y., Tsui T.Y., Pharr G.M. (1997). Elastic properties of human cortical and trabecular lamellar bone measured by nanoindentation. Biomaterials.

[33] Oyen M.L. (2006). Nanoindentation hardness of mineralized tissues. J Biomech.

[34] Porter A.E., Nalla R.K., Minor A., Jinschek J.R., Kisielowski C., Radmilovic V. (2005). A transmission electron microscopy study of mineralization in age-induced transparent dentin. Biomaterials.

[35] Hengsberger S., Kulik A., Zysset P. (2002). Nanoindentation discriminates the elastic properties of individual human bone lamellae under dry and physiological condition. Bone.

[36] Silva M.J., Brodt M.D., Fan Z., Rho J.Y. (2004). Nanoindentation and whole-bone bending estimates of material properties in bones from the senescence accelerated mouse SAMP6. J Biomech.

[37] Rho J.Y., Pharr G.M. (1999). Effects of drying on the mechanical properties of bovine femur measured by nanoindentation. J Mater Sci Mater Med.

[38] Fan Z., Rho J.Y. (2003). Effects of viscoelasticity and time-dependent plasticity on nanoindentation measurements of human cortical bone. J Biomed Mater Res A.

[39] Akhter M.P., Fan Z., Rho J.Y. (2004). Bone intrinsic material properties in three inbred mouse strains. Calcif Tissue Int.

[40] McBride D.J., Choe V., Shapiro J.R., Brodsky B. (1997). Altered collagen structure in mouse tail tendon lacking the alpha(2)I chain. J Mol Biol.

[41] Miles C.A., Sims T.J., Camacho N.P., Bailey A.J. (2002). The role of the [alpha]2 chain in the stabilization of the collagen type I heterotrimer: a study of the type I homotrimer in *oim* mouse tissues. J Mol Biol.

[42] Sims T.J., Miles C.A., Bailey A.J., Camacho N.P. (2011). Properties of collagen in *oim* mouse tissues. Connect Tissue Res.

[43] Kuznetsova N., McBride D.J., Leikin S. (2001). Osteogenesis imperfecta murine: interaction between type I collagen homotrimers. J Mol Biol.

[44] Kuznetsova N.V., McBride D.J., Leikin S. (2003). Changes in thermal stability and microunfolding pattern of collagen helix resulting from the loss of [alpha]2(I) chain in osteogenesis imperfecta murine. J Mol Biol.

[45] Ferguson V.L., Bushby A.J., Boyde A. (2003). Nanomechanical properties and mineral concentration in articular calcified cartilage and subchondral bone. J Anat.

[46] Doube M., Firth E.C., Boyde A., Bushby A.J. (2010). Combined nanoindentation testing and scanning electron microscopy of bone and articular calcified cartilage in an equine fracture predilection site. Eur Cell Mater.

[47] Currey J.D. (1964). Three analogies to explain the mechanical properties of bone. Biorheology.

[48] Hellmich C., Ulm F.-J. (2002). Are mineralized tissues open crystal foams reinforced by crosslinked collagen?—Some energy arguments. J Biomech.

[49] Hellmich C, Barthélémy J-F, Dormieux L. Mineral–collagen interactions in elasticity of bone ultrastructure — a continuum micromechanics approach. European Journal of Mechanics - A/Solids September;23:783–810.

[50] Oyen M.L. (2005). Ultrastructural characterization of time-dependent, inhomogeneous materials and tissues. http://oyenlab.org/research/publication-archives/.

[51] Oyen M.L., Ko C.-C. (2005). Finite element modeling of bone ultrastructure as a two-phase composite. Mechanical properties of bioinspired and biological materials.

[52] Park-Wyllie L.Y., Mamdani M.M., Juurlink D.N., Hawker G.A., Gunraj N., Austin P.C. (2011). Bisphosphonate use and the risk of subtrochanteric or femoral shaft fractures in older women. JAMA.

[53] Marini J.C. (2003). Do bisphosphonates make children's bones better or brittle?. N Engl J Med.

